# Supplementary material for: FERN – a Java framework for stochastic simulation and evaluation of reaction networks
Source: BMC Bioinformatics. 2008 Aug 29;9:356. doi: 10.1186/1471-2105-9-356 (PMC2553347; doi:10.1186/1471-2105-9-356)
Supplement: Additional file 1 — FERN distribution, Version 1.3. This archive contains the FERN source code and binaries as well as documentation and example models in FernML and SBML. [file 1471-2105-9-356-S1.zip › fern/doc/javadoc/fern/network/PropensityCalculator.html]

PropensityCalculator


---


|  |  |  |
| --- | --- | --- |
| **Overview** \| **Package** \| **Class** \| **Use** \| **Tree** \| **Deprecated** \| **Index** \| **Help** | |  |
| **PREV CLASS**   NEXT CLASS | **FRAMES**    **NO FRAMES**     **All Classes** |  |
| SUMMARY: NESTED \| FIELD \| CONSTR \| METHOD | DETAIL: FIELD \| CONSTR \| METHOD |  |


---


## fern.network Interface PropensityCalculator

**All Known Subinterfaces:**: ComplexDependenciesPropensityCalculator

**All Known Implementing Classes:**: AbstractKineticConstantPropensityCalculator, ArrayKineticConstantPropensityCalculator, CellDesignerPropensityCalculator, SBMLPropensityCalculator

---

``` public interface PropensityCalculator ```

A `PropensityCalculator` is the way, a `Network` tells a `Simulator`
how to calculate propensities for the reactions. Each `Simulator` calls the  `calculatePropensity` method, when the amount of some reactants of a reactions has changed.

**Author:**
:   Florian Erhard

---

| **Method Summary** | |
| --- | --- |
| `double` | `calculatePropensity(int reaction, AmountManager amount, Simulator sim)`             Calculates the propensity for a reaction given the amounts of the `AmountManager`. |

**Method Detail**

### calculatePropensity

```
double calculatePropensity(int reaction,
                           AmountManager amount,
                           Simulator sim)
```

:   Calculates the propensity for a reaction given the amounts of the `AmountManager`.
    If a positive
    value for volume is given, it is assumed that the constants are deterministic rate
    constants and are hence to be transformed to specific reaction rate constants.

    :   **Parameters:**: `reaction` - index of the reaction: `amount` - AmountManager: `sim` - Simulator **Returns:**: actual propensity of the reaction


---


|  |  |  |
| --- | --- | --- |
| **Overview** \| **Package** \| **Class** \| **Use** \| **Tree** \| **Deprecated** \| **Index** \| **Help** | |  |
| **PREV CLASS**   NEXT CLASS | **FRAMES**    **NO FRAMES**     **All Classes** |  |
| SUMMARY: NESTED \| FIELD \| CONSTR \| METHOD | DETAIL: FIELD \| CONSTR \| METHOD |  |


---
